# The main tasks of a semiotics of artificial intelligence

**DOI:** 10.1515/lass-2022-0006

**Published:** 2023-03-28

**Authors:** Massimo Leone

**Affiliations:** University of Turin, Turin, Italy; Shanghai University, Shanghai, China; University of Cambridge, Cambridge, England; Bruno Kessler Foundation, Trento, Italy

**Keywords:** fake, generative adversarial networks, simulation, undecidability of the digital fake

## Abstract

The article indicates the essential tasks of a semiotics of artificial intelligence: studying the way it simulates the expression of intelligence; the way it produces content that is creatively endowed; the ideological assumptions of artificial intelligence within the culture that produces it. Artificial intelligence is, from a semiotic point of view, the predominant technology of fakery in the current era. On the strength of its studies on the false, semiotics can therefore also be applied to the analysis of the fake that, in increasingly sophisticated forms, is produced through artificial intelligence and through the deep learning of neural networks. The article focuses on the adversarial ones, trying to highlight their ideological assumptions and cultural developments, which seem to indicate the entry of human societies and cultures into the ‘realm of the absolute fake’.

## 1 The study of simulative artifacts

**Figure 1:**
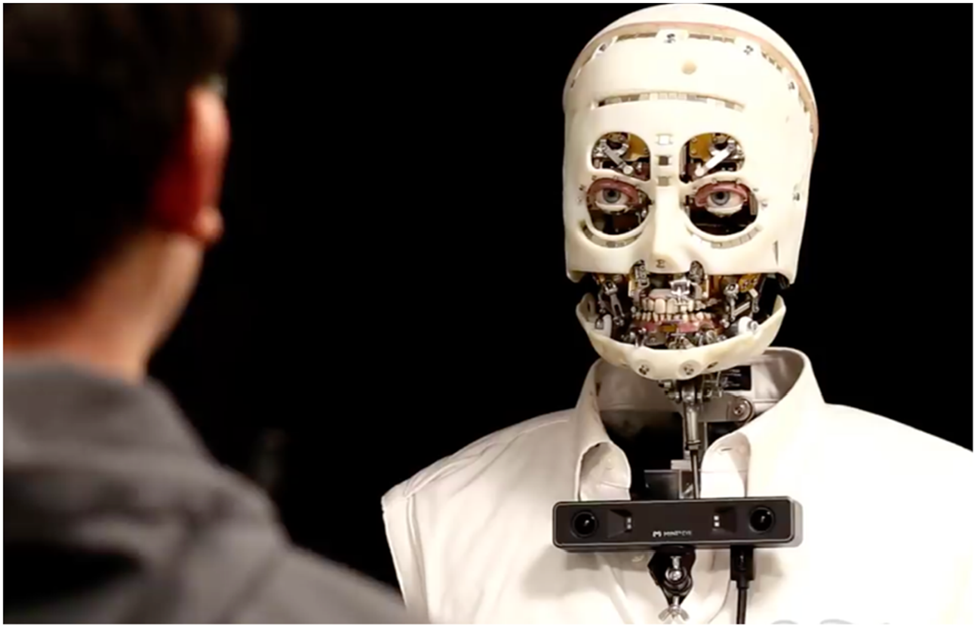
Disney-financed android robot Gaze ‘interacting’ face-to-face with a human being; image in the public domain.

Semiotics should concentrate on studying the efforts to simulate human intelligent behaviors through non-organic and non-human devices. This simulation can take place at the level of expression, at that of content, or at both. At the level of expression, the focus is on the inorganic reproduction of signs that humans associate with intelligence. See for instance, Disney-financed project “Gaze”,1*Gaze*, a robot developed by Walt Disney Imagineering with a team of researchers from the University of Illinois at Urbana-Champaign and the California Institute of Technology, is the result of advanced research in the field of technology and presents a very interesting feature. It can reproduce certain specific expressions of the human face. For example, it can make small movements of the head or to blink its eyelids. a robot that simulates human expressions and, moreover, emulates them in a face-to-face interaction (Figure 1).

In this technology, essentially inorganic matter is arranged, also through artificial intelligence, to convey an impression of understanding. Facial expressions are not only cognitive, but some are, and many are essential to communicate mutual intelligibility. At the level of expression, gigantic steps forward have been made already. That is evident in the domain of verbal language. Expression through verbal language is tightly associated with impressions of intelligence. Non-human animals tend not to appear as endowed with the same intelligence and sensibility as humans because they do not have access to the same language. But verbal language can be simulated at its expression level without necessarily involving a simulation at the content level. To be more precise, artificial intelligence can simulate verbal language through a syntactic mastering of human verbal language. We can speak to our IA speaker, like Siri or Alexa. But can we really? The name itself, “speaker”, reveals that the tendency to attribute human intelligent behaviors to devices is old. In the past, that was mostly metaphoric. A traditional pre-IA speaker was not able to speak or to be spoken to. It just mechanically reproduced sounds from a source. But a new generation speaker connected with artificial intelligence can, to a certain extent, interact with humans through verbal language. Syntactic mastering of verbal language provides a solid infrastructure for the simulation of intelligence. It is, however, also fundamental that this intelligence speaks to us through a human voice.

**Figure 2:**
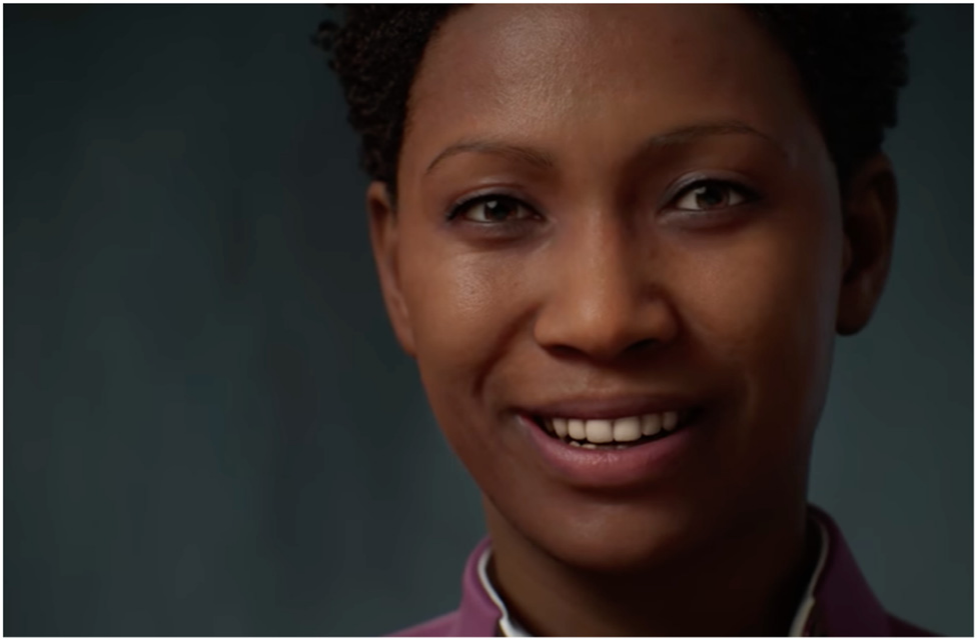
An example of a photorealistic face image created by the MetaHuman browser; image in the public domain.

A semiotics of Artificial Intelligence can apply itself, first, to the study of all those signs that are used to simulate intelligence. At the expressive level, references to the human body are paramount. European Research Council project FACETS concentrates on the digital face because of its current social relevance. An aspect of it concerns artificial intelligence. More and more, simulating the human face is becoming central in simulating artificial intelligence, especially at the expressive level. Simulating an intelligent pragmatics requires simulating an intelligent aesthetics. The phenomenon of the uncanny valley measures the resistance to the aesthetics of simulated faces, but also indicates its power. In the future, simulating an intelligent face will be a central feature of artificial intelligence. The videogame industry, as usual, is ahead. A new *MetaHuman* tool already offers users the ability to create a photo-realistic digital image of a human face inside a browser (Figure 2). Epic Games announced this browser-based software powered by its Unreal Engine.2The company has shared several videos explaining this technology.

In the more distant future, the generation of digital faces might combine with that of biological faces. As Kim et al. 2020 (online) put it:

Regenerating lost or damaged tissue is the primary goal of Tissue Engineering. 3D bioprinting technologies have been widely applied in many research areas of tissue regeneration and disease modeling with unprecedented spatial resolution and tissue-like complexity. However, the extraction of tissue architecture and the generation of high-resolution blueprints are challenging tasks for tissue regeneration. Traditionally, such spatial information is obtained from a collection of microscopic images and then combined together to visualize regions of interest. To fabricate such engineered tissues, rendered microscopic images are transformed to code to inform a 3D bioprinting process. If this process is augmented with data-driven approaches and streamlined with machine intelligence, identification of an optimal blueprint can become an achievable task for functional tissue regeneration.

At a deeper level, Artificial Intelligence is more and more applied to genomics and genetic engineering. Limits to the recreation of specific human faces through AI-enhanced genetic engineering are currently more ethical than technical. Genes can now be edited, as in CRISPR gene editing, a genetic engineering technique in molecular biology by which the genomes of living organisms may be modified.3It is based on a simplified version of the bacterial CRISPR-Cas9 antiviral defense system. By delivering the Cas9 nuclease complexed with a synthetic guide RNA (gRNA) into a cell, the cell’s genome can be cut at a desired location, allowing existing genes to be removed and/or new ones added *in vivo* (in living organisms). For the time being, artificial intelligence and deep learning are rapidly progressing in the simulation of photo-realistic faces. Semiotics must study the rhetoric of artificial intelligence, that is, the expressive signs that simulate an intelligent behavior or device (the face being the human primary intelligence expressive device).

At the level of content, the focus is on the inorganic reproduction of meaning that humans associate with intelligence. The meaning produced by artificial intelligence is, at a first level, behavioristic, that is, humans realize that machines are intelligent because they produce intelligent behaviors. A calculator can produce new output based on known input, yet it would be hardly qualified as an intelligent device. Intelligent behavior must go beyond computation. Its outputs must be synthetic, not simply analytic. In the terms of C.S. Peirce, a machine produces a behavior that can be qualified as intelligent if it involves not only deduction and induction, but also abduction. In this case too, however, the believability of artificial intelligence depends on its aesthetics. An old calculator and a present-day robot are both computational devices, yet the second is endowed with a human-like interface (a voice, a face).

More generally, one must distinguish between simulation of intelligence and proper artificial intelligence. The former is mostly a matter of expressive signs, whereas the latter requires creativity at the level of content. But is artificial intelligence creative? At this level, that of the study of artificial intelligence as meaningful content-producing technology, what matters is to define intelligence, the different kinds of it, and the ways in which they beget creativity. There are several ways in which creative content is created by machine through artificial intelligence (specifically, deep learning). The most common one today is the recognition of pattern in big data. Artificial Intelligence can recognize configurations that are not initially singled out by researchers because of the size of the databases on which it can operate. In the past, the advantage of artificial intelligence on human intelligence was largely computational. Master chess players would still win on machines because they could identify and plan patterns of playing that were ignored by machines. But now the quantity is becoming quality.

In the 1980s, at the height of his career, chess world champion Garry Kasparov claimed that there would never be a chess program capable of defeating him. And indeed, in 1989, he played two games against IBM’s computer Deep Thought, both of which he won. In 1996, Kasparov defeated Deep Thought successor, Deep Blue, in a match over six games with 4:2 but was the first chess world champion ever to lose a game under tournament conditions against a chess program. The following year, Kasparov was defeated by Deep Blue in the rematch. Deep Blue surprised the world with an ‘instinctive’, superior game that seemed creative in many ways. Kasparov spread the rumor that IBM must have cheated. Nowadays, in 100 matches, the reigning world chess champion Magnus Carlsen would not score a single victory against the world’s best chess program. Carlsen currently has an Elo rating of 2,845 (February 2019), while Stockfish 9 has a rating of 3,438 (the engine ratings are not FIDE ratings, but the player pool for engines is much stronger than for humans, so theoretically a FIDE rating for Stockfish 9 would be even higher). The recent performances of AI in typical Chinese game go are even more spectacular. The game of go has long been viewed as the most challenging of classic games for artificial intelligence owing to its enormous search space and the difficulty of evaluating board positions and moves. Silver et al. (2016) introduced a new approach to computer go that uses ‘value networks’4The network which is trained into assigning a determinate output by giving a particular input to the game is known as Policy Network; the value network gives value/score to the state of the game by calculating a foreseen cumulative score for the current states. Every state goes through the value network. The states which get more reward get more value in the network. In other terms, a policy network is used to compute prior probabilities to guide what move the Monte-Carlo search should pick; a value network is used to generate data to validate the policy network. In computer science, a Monte Carlo tree search (MCTS) is a heuristic search algorithm that uses random sampling for deterministic problems which are difficult or impossible to solve using other approaches. to evaluate board positions and ‘policy networks’ to select moves. These deep neural networks are trained by a novel combination of supervised learning from human expert games, and reinforcement learning from games of self-play. Without any lookahead search, the neural networks play go at the level of state-of-the-art Monte Carlo tree search programs that simulate thousands of random games of self-play. Silver et al. (2016) also introduced a new search algorithm that combines Monte Carlo simulation with value and policy networks. Using this search algorithm, the program AlphaGo achieved a 99.8% winning rate against other go programs and defeated the human European go champion by 5 games to 0, this being the first time that a computer program has defeated a human professional player in the full-sized game of go, a feat previously thought to be at least a decade away.

## 2 The study of simulative ideologies

A semiotic-oriented philosophy of digital communication aims at reading the technologies of meaning in the long period of the history of human semiotic systems, to reveal the implicit ideologies that underlie the creation of new devices, processes, and artefacts of meaning. Artificial intelligence is no exception, as its development is usually underpinned by specific preconceptions about what intelligence is, how it should work, and what kinds of results it is supposed to generate in the world. Since artificial intelligence appears to be, at least at this stage of its development, a simulation of human intelligence, semiotics can study it as a particular case of forgery, a theme that semiotics knows well. Each culture and each historical epoch are characterized by the specific semiotic modalities that they adopt in the production of the fake; artificial intelligence is becoming the chief modality in the present-day digital production of fake. The human species is endowed with an innate capacity to give rise to representations that intentionally do not correspond to any ontological reality. The technologies and languages of forgery, however, change in time and space. With digital technology, telematic communication, and, above all, with artificial intelligence and deep learning, the human culture of the fake is crossing a decisive threshold.

In semiotic terms, and in particular in the terms of Greimas’ and Floch’s square of veridiction, verisimilitude can be briefly defined as what seems to be truthful, although it might be not, so that psychologically it gives rise to a condition of doubt. Falsity, instead, describes the character of what lacks correctness and is contrary to the truth, so that there is no doubt; in the terms of the square of veridiction, it is what neither is truthful nor appears as such. Finally, a lie is an affirmation of what is false. Taking up in a detailed way these definitions of verisimilitude, falsity, and lie, would help semiotics understanding the human/digital dialectic considered in this article. Verisimilitude results from the iconic proximity between digital objects and what they represent, meaning that a digital artefact, for instance, is able to represent a face but within a context that leaves us in doubt as whether it is truthful or not; falsity would be rather of the order of unexpected games, for example the capacity to allow surprising objects to deploy capacities, as it is the case with deepfakes: we know that they are fake, yet they surprise us for their ability to simulate a face that does not exist or that did never exist in the way represented by the video; when we watch a deepfake, and we are given sufficient clues about its falsity, we are amused but also worried, for we realize that, given appropriate contextual conditions, a deepfake could work as a perfect simulation to us (or even against us) and lie us into believing what is not.

In the digital world, indeed, human cultures enter the realm of ‘the absolute fake’. This is due, in the first place, to the material characteristics of digital technology: anything that can be the subject of digital representations can also be the subject of digital representations without ontological reference. Any digital image that will be produced of my aged face in a future whose ontology does not exist yet can be reconstructed in the present by a digital simulation. Second, the realm of the absolute fake is caused by the power of quantitative accumulation: an image of my rejuvenated face may circulate on social media so intensely and virally that it will eventually represent my identity on the web. Third, the domain of the absolute fake is caused by its new modes of creation: previously, falsehood was an issue played out between counterfeiters and connoisseurs (for example, in the field of art); now this game is played more and more by algorithms with largely unpredictable results. Artificial intelligence applied to the creation of the fake has always been practiced vis-à-vis a particular object, namely, the face, which is the main interface and the most important human device for interpersonal communication.

Semiotics is perfectly equipped to conduct a study whose object lies at the crossroads between the fake, the face, and digital representations constructed by artificial intelligence. As for the fake, all the founding fathers of semiotics looked into the subject (Ousmanova 2004): (1) Charles S. Peirce in the American tradition (Cooke 2014); (2) the main voices of structural semiotics, from a special issue of the French journal *Communication* devoted to the concept of “vraisemblable”: Tzvetan Todorov, Gérard Genette, Christian Metz, Julia Kristeva, Gérard Genot, Roland Barthes and others (Todorov 1968); Baudrillard returned to the subject (1987, 2000); more recently, a round table on “Post-Truth and Democracy” was organized by Jacques Fontanille during the Congress of the French Association for Semiotics in Lyon, June 11–14, 2019 (Di Caterino 2020); Umberto Eco has written extensively on forgery (1975, 1984, 1995), edited a special issue of the semiotic journal *Versus* on “Fakes, Identity, and the Real Thing” (1987; with essays by Eco, Prieto, Calabrese, et al.), and has also dealt with the subject in numerous essays and novels (*Il pendolo di Foucault*, *Il cimitero di Praga*, *Numero Zero*); (3) Jurij M. Lotman has repeatedly addressed the issue of the fake (Andrews 2003, p. 101; Makarychev and Yatsyk 2017). The face too has been the subject of research since the beginning of modern anthropology (Leone 2021). In semiotics, after Patrizia Magli’s books on physiognomy, the ERC FACETS project has consistently developed the literature on this subject.

## 3 Case study: the faces of artificial intelligence

Semiotic research on digital representations of the face is growing more and more, especially regarding the representation of the face by artificial intelligence. To develop an analysis of the semiotic ideologies that underlie the creation of synthetic faces, one must however look at the origin of the algorithms which, in recent years, have revolutionized practices in this field. One must return to their founding text, an article that the young Ian J. Goodfellow published on June 10, 2014 — when he was a doctoral student at the University of Montreal — with the title *Generative Adversarial Nets*. Since then, this researcher has become a world guru of artificial intelligence and especially deep learning, holding top positions in the field, among which that of director of the machine learning department in Apple’s Special Projects Group.

Together with a group of friends who were PhD students in computer science, Ian J. Goodfellow proposed a new framework for estimating generative models via a contradictory process, in which two models are trained simultaneously: a generative model, which captures the distribution of data, and a discriminative model, which estimates the probability that a sample comes from the training data rather than from the generative model. The generative antagonistic model has led to groundbreaking applications in artificial intelligence and deep learning, including the creation of ‘artificial faces’ (Leone 2021) and deepfakes. Semiotics has already been applied to the study of artificial intelligence. Yet, it has looked at its results and products, when it would be essential to examine, through semiotics, its ideological presuppositions, and the underlying structure of its functioning.

The production scheme of artificial intelligence imagined by Goodfellow consists in an opposition between two instances; the framework of structural semiotics can therefore contribute to its intelligibility. Two main actants appear in the abstract architecture of GANs. The first is an actant generator that examines a data configuration and produces a text which could be issued from this very configuration; the second is an actant discriminator that examines the text thus produced and evaluates whether it comes from the data configuration or from the generating actant. From an epistemic point of view, therefore, the generating actant aims ‘to make appear’ and therefore ‘to pass as true’ what is not, while the discriminatory actant aims ‘to make appear’ and therefore ‘to unmask as false’ what is not true.

**Figure 3:**
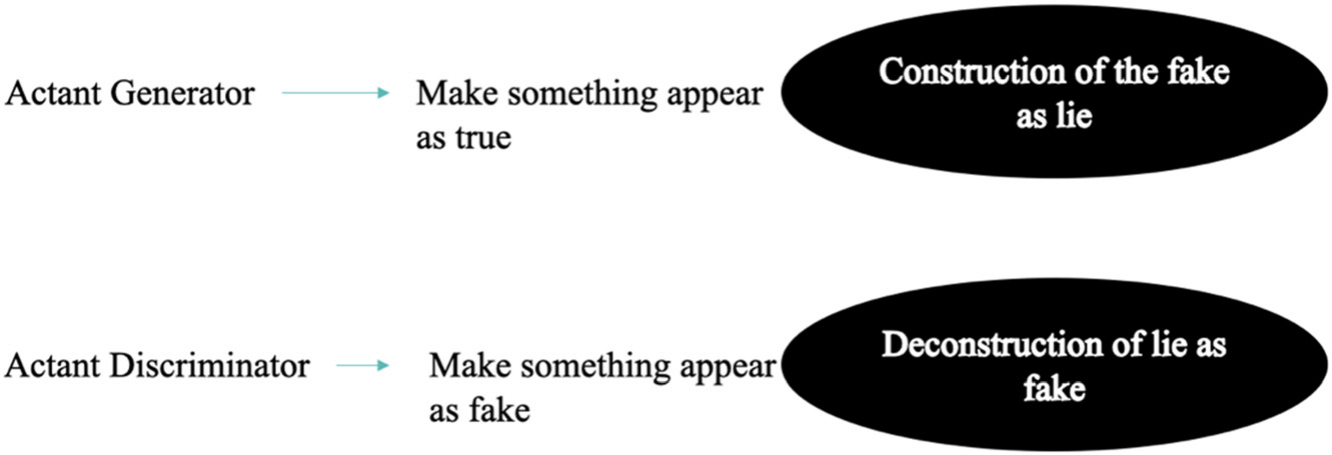
The actantial dialectic in adversarial neural networks.

In mathematical terms, to learn the distribution of the generator pg on the data x, an a priori on the input noise variables pz (z) is defined, then a mapping to the data space as G is represented (z; θg), where G is a differentiable function represented by a multilayer perceptron with parameters θg. A second multilayer perceptron D (x; θd), which produces a single scalar, is also defined. D (x) represents the probability that x comes from the data rather than from pg. D is formed to maximize the probability of assigning the correct label to both the training examples and the samples of G. Simultaneously, G is formed to minimize log(1 - D (G (z))) (Figure 3).

**Figure 4:**
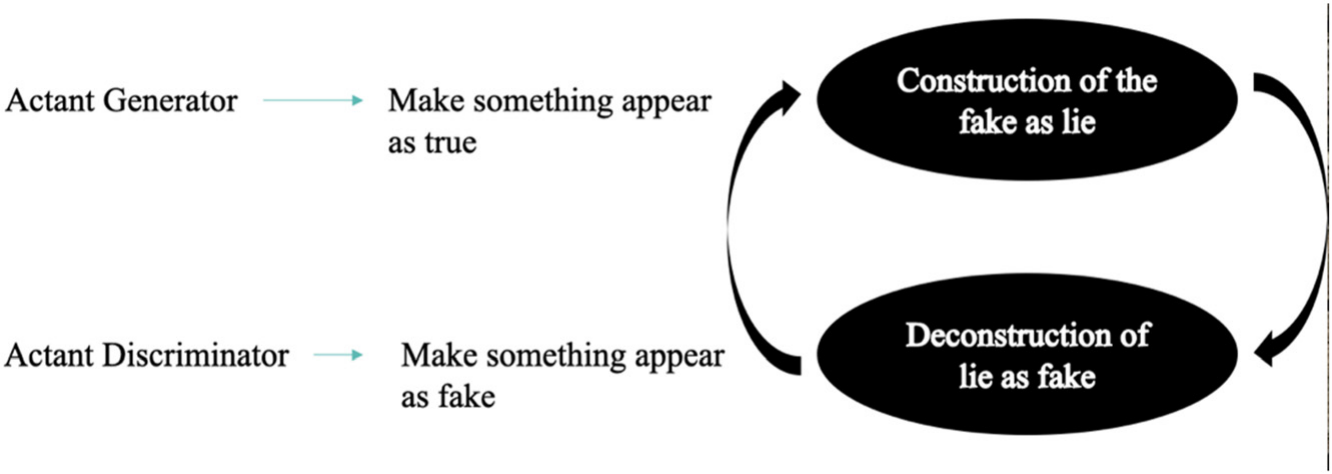
The actantial spiral in adversarial neural networks.

The discriminating actant (D) is at the same time the anti-subject of the generating actant (G), its receiver, and its adjuvant. D sanctions G’s products, designating them as true or false (that is to say, as proceeding from the dataset or not); when the sanction is positive, however, it also *ipso facto* determines the defeat of D with respect to its anti-subject G, and vice versa: when the sanction is negative, this leads to the defeat of G with respect to its anti-subject D. Yet in any case D is always also the adjuvant of G, because the second learns from each sanction of D how better to deceive its counterpart (Figure 4).

When one reads, through semiotics, the founding article “Generative Adversarial Nets” (GAN), one is struck above all by two elements: (1) the conception of artificial intelligence that it expresses is based on the idea of antagonism (neither cooperation, nor mere competition); (2) the metaphor that best explains the new deep learning architecture is that of the forger and the connoisseur (especially in the manufacture of money). Both aspects deserve further philosophical and semiotic reflection, because this new architecture of artificial intelligence now finds application in many professional and social fields, and in particular in the creation of images and synthetic videos of static or moving faces, more and more associated with heads, with bodies, as well as with synthetic contexts, and often expressing themselves through multiple systems of signs, such as facial expressions, gestures, movements, fragments of verbal speech, songs, dances, etc.

The diagram of GANs can be read through the metaphor proposed by the same Goodfellow in 2014: D and G behave like a connoisseur and a forger, respectively. The forger examines the currency in circulation and tries to produce fake samples of it; the connoisseur examines the currency produced by the counterfeiter without knowing its origin and tries to understand whether it is counterfeit or genuine money. In doing so, however, the connoisseur gives information to the forger which will be useful in creating counterfeit money that is even more difficult to distinguish from the genuine one. But the connoisseur also learns to discriminate better and better between authentic and counterfeit money. The metaphor of the art market can also convey the idea of this spiral of generation and discrimination: a forger tries to put fake Modiglianis into circulation, while a connoisseur tries to distinguish them from genuine Modiglianis, but so doing, the latter provides the former with information on how to better falsify works; vice versa, the former also learns from the latter how to falsify the works of the Italian artist.

One must wonder about the nature of the observing actant of this spiral. The products of the generator model are in fact not sanctioned only by the discriminator model but also by a human receiver, who coincides, at least in the first instance, with the receiver of the GANs. Models are programmed by a human sender, yet their ‘behaviors’ are not entirely predictable, not least due to the computational mismatch between human cognition and artificial intelligence. The human programmer is therefore both the sender and the receiver of the products of the interaction between the generator model and the discriminator one. In addition, beyond this professional observer actant, there is another which is made up of those who will receive the products of the generator model without knowing their origin. The spiral that has just been described is intended to increase more and more the epistemic uncertainty of this nonprofessional observer actor.

To say it in simpler terms in the context of the first metaphor: the competition between counterfeiter and connoisseur puts into circulation money or works of art which are fake, but which are increasingly difficult to recognize as such, especially by the observing actant lying outside the spiral. The massive circulation of a fake that is no longer identifiable as such ends up casting epistemic discredit also regarding authentic works of art, and authentic currency. In this resides, perhaps, the most important danger of the ‘spiral of falsehood’. Some researchers have shed a positive light on GANs, suggesting that their internal dialectic should rather be compared to that between teacher and student. The generator model would therefore be like a student trying to produce credible representations from a dataset, while the discriminator model would be like a teacher examining and evaluating these representations. This is partly true, but what makes the difference is that, in the world of GANs, representations of the generator model begin to circulate without reference to the learning context.

That is also the difference between the digital and the analogic fake. The human species is intrinsically capable of intentionally producing false representations of reality, namely representations which, while devoid of indexical origin, simulate one by creating an iconic meaning effect. This ability was probably selected by the species’ biological evolution as adaptive, as it enabled humans to mentally experience potentially dangerous situations without having to experience them empirically. It also enabled them to protect themselves from predators or to trap prey. This is an ability that is not absent in other species, both plant and animal. One of the most remarkable peculiarities of lyrebirds, for example, is their ability to imitate sounds, such as those of other birds and various natural elements but also those of the human environment such as the triggering of a camera, a chainsaw, a fire alarm, a hydraulic cylinder, etc.

In the human species, however, this ability, expressed in and through language, has given rise to a sort of exaptation, consisting in the ability to attach aesthetic pleasure and value to intentionally false representations, which triggered in turn a huge production of fictional texts. Digital technology introduces an essential qualitative and quantitative change in the history of the relationship of the human species with the false. The digital is endowed with a protean materiality whose semiotic manifestation is fully programmable, which is never the case in the manifestation of pre-digital texts. That implies that any digital representation having an indexical relation to its object can be reproduced identically even when this relation is absent; painting can, of course, simulate faces that do not exist, and yet the gap between the ontological face and the painted one will always be obvious, which is not the case in the digital. This absorbs the sense of indexicality which is characteristic of photography and reproduces it in the absence of indexicality; at the same time, it introduces full programmability into the construction of the photographic image. The painting can represent non-existent objects but cannot make one believe in their existence; analogic photography can make people believe in the existence of the objects it represents, but it cannot represent non-existent objects, at least not effectively; digital photography can make people believe in the existence of the nonexistent objects it represents.

## 4 Conclusions

The application of artificial intelligence, and in particular deep learning by GANs, to the production of the material manifestation of digital representations removes them from human evaluation. The fake is inseparable from the human species, yet it is the first time in the history of the species that non-human agents have been put in the condition of producing a fake whose evaluation increasingly escapes humans and is entrusted more and more to an examination that is in turn carried out by means of artificial intelligence. Digital fakes can now reproduce and circulate with unprecedented ease, and this quantitative aspect also results in a qualitative change: it is as if authentic art had to defend itself from an infinite number of counterfeiters who work unceasingly and very quickly in the production of copies.

The digital fake is destined, in the long run, to be indistinguishable from the ‘digital real’; in the case of faces, for example, it is only a matter of time before one can no longer know from the digital photo of a face whether the photo was produced from a biological and ontological face or whether it is a synthetic image. Semiotics tends to problematize the logical concept of truth as an adequacy to the real, by considering, rather, the semiotic conditions that produce a ‘reality effect’. Yet, explaining the rhetoric of a reality effect without postulating an ontological reality leads to inescapable aporias. Similarly, one can well problematize the reality effect of an analogic photograph, but one must also recognize that the arrival of the digital, and of digital deep learning applied to the creation of images, undermines the possibility of distinguishing between a referential image endowed with an effect of reality and a synthetic image producing the same effect.
